# Dietary Patterns of Healthy Underweight Individuals Compared to Normal-BMI Individuals Using Photographic Food Diaries

**DOI:** 10.3390/nu16213637

**Published:** 2024-10-25

**Authors:** Ying Yu, Zhengjie Zhang, Xinrui Gao, Sumei Hu, John R. Speakman

**Affiliations:** 1 Shenzhen Key Laboratory of Metabolic Health, Center for Energy Metabolism and Reproduction, Shenzhen Institutes of Advanced Technology, Chinese Academy of Sciences, Shenzhen 518055, China; y.yu@siat.ac.cn; 2 Health Sciences Institute, China Medical University, Shenyang 110122, China; zsy1883476@163.com; 3 Beijing Engineering and Technology Research Center of Food Additives, National Soybean Processing Industry Technology Innovation Center, Beijing Technology and Business University, Beijing 100048, China; xr.gao@siat.ac.cn; 4 State Key Laboratory of Molecular Developmental Biology, Institute of Genetics and Developmental Biology, Chinese Academy of Sciences, Beijing 100101, China; 5 School of Biological Sciences, University of Aberdeen, Aberdeen AB24 3FX, UK

**Keywords:** healthy underweight, dietary patterns, photographic food diaries

## Abstract

Background: Previously, we found that healthy underweight (HU) subjects, with BMI < 18.5, eat about 12% less food (by calories) each day. It is presently unclear whether this lower intake is associated with them making food choices that provide high satiation and satiety. Methods: Using 7-day photographic records of food intake, we analyzed 52 HU and 50 normal-weight participants. Results: We included 52 HU and 50 normal-weight participants in the final analysis. HU individuals ate 25% fewer calories than normal-weight individuals. Their intake included a higher % of rice (*p* = 0.0013) and vegetables (*p* = 0.0006) and a lower % of livestock meat (*p* = 0.0007), poultry meat (*p* < 0.0001), and starchy roots (*p* = 0.0015), compared with the normal-weight population. The percent energy from carbohydrates was significantly higher (*p* = 0.0234), and the % energy from fat was significantly lower (*p* < 0.0001) in the HU group, with no difference in the % energy from protein. HU individuals sourced more of their protein from plants. Dietary patterns were grouped into three clusters, with 24 individuals grouped into cluster 1 (87.5% normal-weight population), 28 individuals into cluster 2 (64.3% normal-weight group), and 50 individuals into cluster 3 (78% HU group). Conclusions: The HU group ate less overall and had proportionally more rice and vegetables and less poultry and livestock meat, starchy roots, and drinks. With respect to macronutrients, they also ate a greater % carbohydrates and less % fat, and they sourced more of their protein intake from plant sources. HU individuals did not follow a low-carbohydrate lifestyle.

## 1. Introduction

Although the rates of obesity have dramatically increased over the past 70 years in many western countries [1], the distribution of body mass index (BMI) is also becoming increasingly skewed towards the right [2]. This reflects the fact that there is a persistent group of people, comprising about 3–6% of the total population, who sustain an extremely lean phenotype, with BMIs of less than 18.5 kg/m^2^. This group includes some people who have wasting due to chronic illnesses, such as cancer [3], and individuals with eating disorders, such as anorexia nervosa [4]. Nevertheless, many of them show no signs of illness, a group we have previously termed the “healthy underweight” (HU) population [5]. Elsewhere, they have been called individuals with “persistent thinness” [6] or “constitutional thinness” [7,8]. Because these individuals remain very thin, despite being exposed to the same “obesogenic” environment as individuals living with obesity, understanding the diverse aspects of their energy balance may provide clues to their abilities to regulate their body weight so effectively. It has been suggested that this may potentially provide insights into what goes wrong in others who, in the same environments, develop obesity [9].

Previously, we focused on aspects of the energy expenditure and physical activity of HU individuals [5]. We found that HU subjects were less active than normal-weight individuals but had higher-than-anticipated resting energy expenditure from their body composition, related to greater thyroid activity. Overall, they expended about 12% less total energy per day as measured by doubly labeled water (DLW). On average, if individuals are in energy balance during a DLW measurement, total energy expenditure equals total intake. Hence, the implication of these DLW data is that they eat about 12% less food calories each day. This contrasts suggestions that the food intake of HU individuals is “normal” [8], based on more subjective measurements, such as self-reported food diaries and 24 h recall. It also disproves the myth that these very lean individuals eat large amounts of food that they are then able to burn off with excessive exercise. It is interesting whether this lower intake stems from them making food choices that provide high satiation and satiety—for example with the so called ‘ketogenic’ or low-carbohydrate diets. In the current paper, we explored this question by surveying photographic records of the food intake of HU compared to normal-weight subjects.

## 2. Materials and Methods

### 2.1. Study Design and Participants

In this study, we used the international standard for the classification of underweight, normal weight, overweight, or obesity. Our inclusion criteria were BMI 15–18.5 kg/m^2^ for the healthy underweight group and 21.5–25 kg/m^2^ for the normal-weight group. The interval of BMI range of both groups was 3.5. To maximize the difference between both groups, we chose the higher end of the BMI range for the normal-weight group. We tracked the dietary intakes of Chinese individuals living in Beijing, China. Eligibility criteria included the following: (1) body mass index (BMI), normal-weight individuals (normal) BMI ≥ 21.5 and <25 kg/m^2^, healthy underweight individuals (HU) BMI > 15 and ≤18.5 kg/m^2^; (2) age between 20 and 40 years old. Participants were excluded if they had (1) any metabolic diseases; (2) recent weight loss due to various disease causes; (3) ongoing treatment for weight loss; (4) eating disorders; or (5) were pregnant or lactating women. The sex, age, height, and weight of the participants were collected through questionnaires before the trial. After signing informed consent, all participants were asked to record their diets for consecutive 7 days (no less than four days of recording were required to be considered as valid data).

The trial was reviewed and approved by the Institutional Review Board, Institute of Genetics and Developmental Biology, Chinese Academy of Sciences. The approval code was IGDB-2017-IRB-001. It was registered as clinical trial NCT03221322.

### 2.2. Photographic Food Records

**Figure 1:**
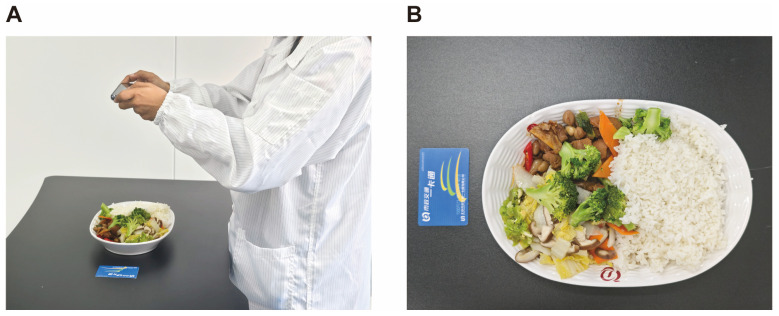
Photographic food records. (**A**) Participant faced the food and took the photograph vertically directly above the food. (**B**) The photograph for recording.

Participants were asked to use the photo function on their cell phones to record all the food and beverages they consumed in a one-week period. To ensure a representative sample was recorded, participants were asked to collect data on at least 4 valid days of records, including three weekdays and one weekend day. Photographs were taken before and after eating each meal or food item, with a standard-sized credit card (8.5 cm × 5.4 cm) by the side of the plate, to provide a scale to enable the subsequent estimation of food portion weights. Participants were required to face the food and take the photograph vertically directly above the food to ensure that the type and portion size of each food item could be recognized (Figure 1).

### 2.3. Food Recognition and Analysis

According to the Chinese Dietary Guidelines, Chinese foods were classified into 16 groups, including rice, flour, coarse food grains, beans, starchy roots, vegetables, fruits, fungi and algae, livestock meat, poultry meat, aquatic products, eggs, milk and milk products, soy products, nuts and seeds, and drinks. A trained professional dietician (YY) was responsible for recognizing the food types and estimating portion weight in the valid photos. According to the collected images of food before and after eating, food was recognized for classification, and the portion size of each food item was estimated with the standard-size card as a reference in the photograph. Then, the energy and macronutrients composition of the food items were calculated according to the China Food Composition (6th Edition) and Boohee app (version 12.2.0, https://www.boohee.com/, accessed on 15 March 2024). Satiety was calculated from the Hava app (version 1.15.3, https://www.hava.co, accessed on 1 August 2024), and the satiety score was determined by four key factors: the protein percentage of the food, the energy density, the fiber content, and the hedonic factor. The schematic overview of food recognition and portion size estimation is shown in Figure S1.

### 2.4. Validation Test of the Food Portion Size Estimation

A validation test was conducted to assess the accuracy of the food portion weight estimation. Ten individuals were instructed to collect five different items of food onto a single plate, selected from a buffet providing around 20 different available foods. Hence, the plates generated in the test contained a diversity of food types and quantities. Photos of these plates of food were taken following the same procedure as the participants in the main study. The actual weight of each item on the plate was weighed by a different person from the person performing the rating of the portion sizes from the photographs. The weights of each food item on each plate were estimated from the photos by the dietitian. Bland–Altman and correlation analyses were then performed between the actual and estimated weight to evaluate the accuracy and biases of the rating procedure.

### 2.5. Redundancy Analysis

As a multivariate method to study linear relationships between two or more matrices, redundancy analysis (RDA) was performed to calculate the variation explained by different factors among dietary and physiological parameters in different groups [10]. Specifically, we used RDA conditioned on covariates to estimate the differences between HU and normal-weight individuals’ variation, explained by dietary and physiological parameters. The explanatory variables were age, height, weight and BMI, the response variables were the weights of the different food items consumed. RDA was performed using the RDA function of the R package vegan [11]. For all RDA models in this study, we carried out 999 permutations to test the significance of the explanatory variables with the R function envfit.

### 2.6. K-Means Clustering

To classify the dietary patterns of different populations, unsupervised clustering of all dietary information was performed using the k-means algorithm. In this study, the fviz_nbclust function from the R package factoextra (Elbow Method) was used to determine the k-value [12]. The result of the elbow method is shown in Figure S2, and there is a variation in slope from steep to shallow (an elbow, k = 3) in discovering the best number of clusters. The k-means clustering was performed using k = 3 with the widely used parameters nstart = 10 and iter.max = 1000.

### 2.7. Statistical Analyses

Sample size calculation was based on total energy expenditure (TEE) from the DLW measurements reported in our previous paper [5]. TEE is the daily total energy expenditure of the whole body, which is considered to be balanced with energy intake at constant body weight. From a previous study, the mean TEEs of the normal-weight group and HU group were 8.82 MJ and 7.75 MJ, respectively, with the standard deviation between the two groups being 1.40 MJ, with which the effect size was calculated to be 0.76. To obtain 95% power and 5% type 1 error, at least 38 participants in each group were required to complete the study (calculated using G*Power v3.1.9.7). In this study, there were 50 people in the normal-weight group, 52 people in the HU group, and the power was 98.56%.

Statistical analysis was performed using R (version 4.3.0) and GraphPad Prism software (version 8.4). The unpaired two-tailed Student’s *t*-test was used to determine the statistical significance of differences between the two groups. For comparisons involving more than two groups, two-way analysis of variance (ANOVA) was used. The statistical analyses and sample size applied for each experiment are indicated in the figure legends. *p* < 0.05 was considered to be statistically significant. Different significant levels were labeled as * *p* < 0.05, ** *p* < 0.01, and *** *p* < 0.005, respectively.

## 3. Results

### 3.1. Baseline Characteristics of Participants

**Figure 2:**
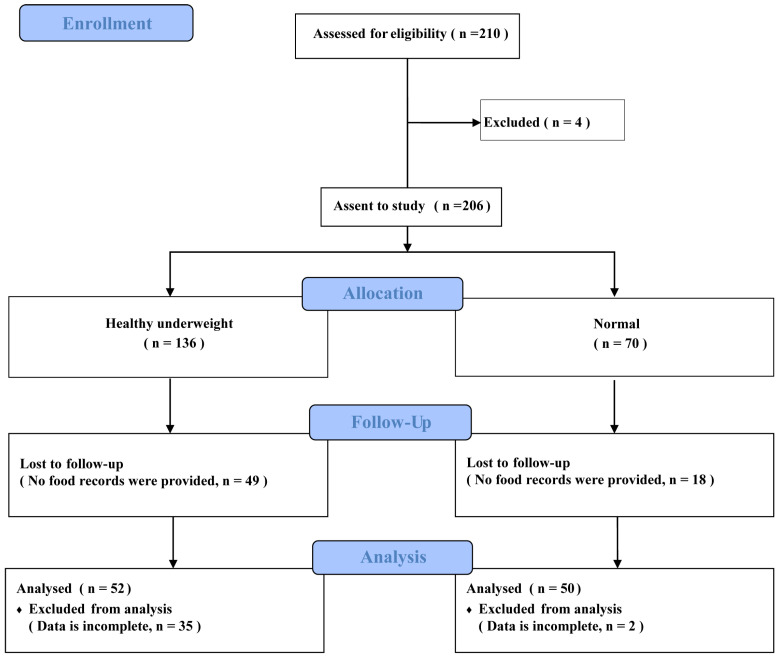
Flowchart of the participants included in this study. Flowchart describing the number of participants throughout the process of enrolment through to completion.

In this study, recruitment and follow-up numbers are summarized in the flowchart in Figure 2. There were 210 participants who attended the screening after responding to an online questionnaire. Four participants were excluded for not meeting the recruitment criteria, resulting in 206 individuals participating in the study, with 136 participants in the HU group and 70 participants in the normal-weight group. In total, 49 individuals in the HU group and 18 individuals in the normal-weight group were excluded as they did not provide any food photographs, with another 35 individuals in the HU group and 2 individuals in the normal-weight group excluded for providing incomplete records (less than 4 days data). There were 52 HU participants and 50 normal-weight participants included in the final analysis. These individuals were paid RMB 100 for participation in the study.

The characteristics of the participants are shown in Table 1. There were 41 females and 11 males among the 52 participants in the HU group, and 35 females and 15 males among the 50 participants in the normal-weight group. The overall groups did not differ significantly in age and height (*p* > 0.05) (Table 1). The BMIs of the HU and normal-weight group were 17.1 ± 1.0 kg/m^2^ and 22.7 ± 1.0 kg/m^2^, respectively (*p* < 0.05).

### 3.2. Validation of the Food Portion Size Estimation

Comparing the estimated values to the actual values in the validation study, the coefficient of variation (CV) was less than 15% for each food item weighed separately and less than 5% for the whole meal weight (Supplemental Table S1). In the Bland–Altman plot for the difference between the actual and estimated values, most of data points fell within the 95% limits of agreement, demonstrating strong agreement between the actual and estimated values. This suggests that the estimation method is reliable (Supplemental Figure S3A). The correlation plot for actual and estimated values in food portion sizes demonstrated a high correlation coefficient (individual: R^2^ = 0.9595, *p* < 0.0001; whole meal: R^2^ = 0.9928, *p* < 0.0001), indicating the high accuracy of the estimates by the dietitian (Supplemental Figure S3B).

### 3.3. Food Recognition and Portion Size Estimation

**Figure 3:**
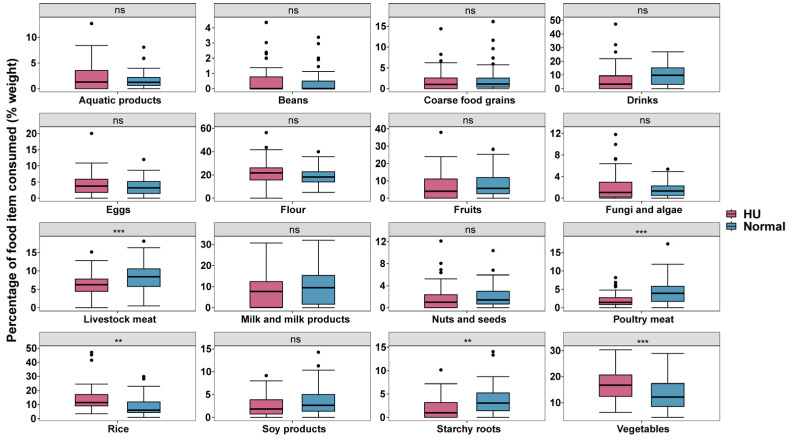
Average % weight of different food items consumed in the daily diet of HU and normal-weight groups. Statistical analysis was performed using unpaired two-tailed Student’s *t* test. ns, *p* > 0.05; **, *p* < 0.01; ***, *p* < 0.001.

On average, HU individuals ate 85.2 ± 54.5 g rice per day, compared with 73.0 ± 54.4 g rice in normal-weight individuals. Although there was no difference in the weight of the food consumed (Table 2), the proportion to the total weight was significantly higher in the HU group compared to the normal-weight group (13.8% vs. 8.5%, *p* = 0.001). For vegetables, the HU individuals ate 110.3 ± 52.2 g (17.2%) and the normal-weight individuals ate 117.6 ± 59.1 g (13.1%), and the proportion was significantly higher in HU individuals (*p* < 0.001). HU individuals ate 39.9 ± 22.3 g of livestock meat, 13.6 ± 14.5 g poultry meat, and 11.2 ± 12.7 g starchy roots, while normal-weight individuals ate 77.2 ± 34.7 g, 39.0 ± 29.6 g, and 30.8 ± 26.1 g, respectively, which were significantly lower in the HU group than the normal-weight group for all three foods, both in terms of the weight of the food consumed and the proportion of the food consumed (Figure 3 and Table 2). Regarding the frequency of food intake, HU individuals ate rice on 37.1% of days compared to 28.6% for normal-weight individuals (*p* = 0.008), while normal-weight individuals consumed drinks (8.1% vs. 17.0%, *p* < 0.001), poultry meat (8.0% vs. 16.1%, *p* < 0.001), soy products (10.7% vs. 15.3%, *p* = 0.017), and starchy roots (6.5% vs. 12.8%, *p* < 0.001) more frequently. There was no significant difference in the frequency of intake of the other foods (Figure S4).

### 3.4. Energy Intake and Macronutrient Composition

**Figure 4:**
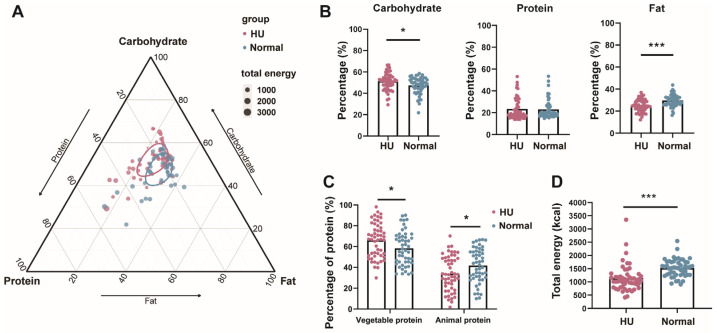
Energy intake and nutritional composition of the diets in HU and normal-weight group. (**A**) Ternary plot of energy supply of three macronutrients. Pink dots represent HU group and blue dots represent normal-weight group. The bigger the dots, the higher the energy intake. Ellipses represent a 95% confidence interval assuming a distribution. (**B**) Differences in energy from carbohydrate, protein, and fat between HU and normal-weight groups. (**C**) Differences in plant protein and animal protein between HU and normal-weight groups. (**D**) Differences in total energy intake between HU and normal-weight groups. Statistical analysis was performed using unpaired two-tailed Student’s *t* test and two-way ANOVA test. *, *p* < 0.05; ***, *p* < 0.001.

Energy intake and nutritional composition were significantly different between the HU group and normal-weight group (*p* < 0.05) (Figure 4 and Table S2). Protein contributed 23.6 ± 9.8%, carbohydrates 51.5 ± 8.0%, and fats 24.6 ± 6.1% of the total energy in the HU group, compared to 23.0 ± 8.8%, 47.4 ± 7.9%, and 29.6 ± 5.3%, respectively, in the normal-weight group. In comparison to the normal-weight group, the % energy from carbohydrates was significantly higher (*p* = 0.023) and the % energy from fat was significantly lower (*p* < 0.0001) in the HU group, with no difference in the % energy from protein (Figure 4A,B and Table 3). However, the sources of the protein intake were different between the two groups, with more plant proteins (65.9% vs. 58.3%, *p* < 0.05) and less animal proteins (34.1% vs. 41.6%, *p* < 0.05) in the HU group than the normal-weight group (Figure 4C). The total daily energy intake of the HU group was 388 kcal lower than the normal-weight group, approximately 25% lower (Figure 4D). Additionally, there was no difference in the predicted satiety of the consumed diets between the HU group and normal-weight group (*p* = 0.2008) (Figure S5). We predicted the energy expenditure for each individual using an equation based on DLW measurements (Bajunaid et al. [13]). There was a strong correlation between the estimated daily energy intake of the participants and the predicted expenditure by DLW (r^2^ = 0.2939, *p* < 0.001) (Figure S6). However, the estimated energy intakes were on average lower than the prediction for both groups. The estimated energy intake of the HU population was 615.1 kcal (39.3%, *p* < 0.001) lower than predicted, and the estimated energy intake of the normal-weight group was 350.8 kcal (19.1%, *p* < 0.001) lower than predicted.

The energy intake and nutritional composition consumed at different times of the day, at breakfast, lunch and dinner, were analyzed separately (Table 3). The HU group had a significantly lower energy intake at breakfast (212.0 ± 125.0 vs. 407.0 ± 190.3, *p* < 0.001), lunch (458.8 ± 293.8 vs. 582.9 ± 173.6, *p* = 0.011), and dinner (467.6 ± 190.2 vs. 560.8 ± 160.9, *p* = 0.009) than the normal-weight group. For breakfast, there was no significant difference in the energy intake from carbohydrates, protein, or fat (*p* > 0.05) between the two groups. For lunch, the HU group had significantly higher energy from carbohydrate (52.9 ± 12.1 vs. 45.8 ± 9.5, *p* = 0.001) and significantly lower energy from fat (23.5 ± 7.6 vs. 30.7 ± 6.4, *p* < 0.001) than the normal-weight group, with no difference in the energy intake from protein (*p* > 0.05). For dinner, the energy intake from fat was significantly lower in the HU group than in the normal-weight group (24.4 ± 7.7 vs. 29.8 ± 8.4, *p* = 0.001), without differences in the energy intake from carbohydrate or protein (*p* > 0.05).

### 3.5. Dietary Patterns of HU and Normal-Weight Groups

**Figure 5:**
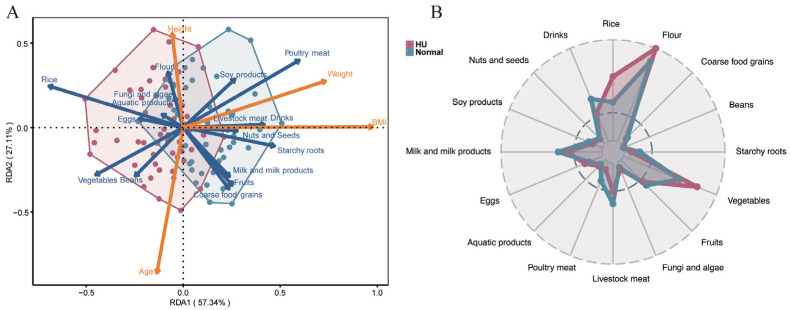
Characteristics of the dietary patterns of HU and normal-weight groups. (**A**) RDA of food items and physiological factors on different dietary patterns. Dark blue arrows represent food items and orange arrows represent physiological factors. Orange arrows represent physiological factors, and blue arrows represent dietary factors. (**B**) Average weight percentage of food consumed by the HU and normal-weight groups.

To calculate the proportion of variation explained by different factors among dietary and physiological factors in both groups, RDA was performed. In RDA, RDA1 and RDA2 usually refer to the first two principal axes which explain the variance in the data associated with the explanatory variables under consideration. In this study, RDA1 explained 57.3% of the variance while RDA2 explained a further 27.1% of the variance. Overall, the two axes explained 84.5% of the variance in the response data, indicating a high association between the response and the explanatory variables. The vectors in the figure reflect the explanatory variables, and their length and direction indicate the correlation with the response data. In the HU and normal-weight groups, BMI was the key physiological determinant influencing dietary patterns (Figure 5). Variations in dietary patterns showed a strong response to BMI. Of these food items, rice intake was positively associated with the dietary pattern of the HU group (Figure 5). Poultry meat intake was positively associated with the dietary pattern of the normal-weight population (Figure 5A). Among the food items assessed, poultry meat, rice, livestock meat, starchy roots, and vegetables had the most significant impact on the separation of dietary patterns. The dietary pattern of HU group was characterized with more rice, more vegetables, less poultry meat, less livestock meat, less starchy roots, and less drinks, in comparison with the normal-weight group (Figure 5B and Figure S7).

### 3.6. Clustering of Dietary Patterns

**Figure 6:**
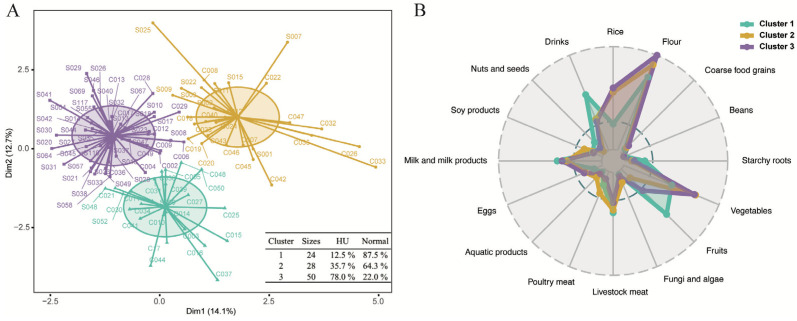
Clustering of dietary patterns using k-means algorithm. (**A**) The characteristics of different clusters, with 24, 28, and 50 individuals grouped to cluster 1, 2, and 3, respectively. (**B**) Average weight percentage of consumed food items in different clusters. Green lines represent cluster 1, orange lines represent cluster 2, and purple lines represent cluster 3.

Unsupervised clustering was performed by the k-means algorithm to investigate the clustering of dietary patterns among all participants. Dietary patterns were grouped into three clusters based on the elbow method, which showed that three clusters provided a significant reduction in the sum of squared distances without diminishing returns in clustering quality (Figure 6). There were 24 individuals grouped into cluster 1, 87.5% of which were of the normal-weight population; 28 individuals into cluster 2, with 64.3% from the normal-weight group, and 50 individuals into cluster 3, with 78% from the HU group (Figure 6A). RDA was conducted to elucidate the dietary patterns within these three distinct clusters. Cluster 1, consisting predominantly of normal-weight group participants, displayed a dietary preference that included a higher consumption of fruits, starchy roots, livestock meat, and drinks. Cluster 2 presented an intermediate dietary pattern between the composition of cluster 1 and 3. Cluster 3, which was primarily composed of individuals from the HU group, was characterized by a dietary pattern that favored a higher intake of rice, flour, and vegetables (Figure 6B and Supplemental Figure S8).

## 4. Discussion

There are few studies on healthy underweight populations. Studying HU populations may provide insights into the treatment and prevention of obesity. Obesity and weight gain results from a long-term positive energy balance [14], at least partly driven by the overconsumption of energy [15]. It is often suggested that characteristics of the environment drive this over consumption, such as availability and resultant excessive intake of ultra-processed foods [16], or foods with high caloric density [17]. Underweight individuals appear able to successfully navigate this obesogenic environment and avoid weight gain. Previously, we reported 12% lower daily energy expenditure in the HU population than the normal-weight population from DLW measurements, which implies a 12% lower energy intake in HU individuals if they were in energy balance when measured. Both the physical activity level (PAL) (1.37 ± 0.23) and PAEE (1.44 ± 1.12 MJ/day) of the HU individuals were significantly lower than the normal-weight individuals (PAL, 1.59 ± 0.23; PAEE, 2.34 ± 1.02 MJ/day) [5]. How HU individuals avoid overconsumption may depend on their diet choices—favoring food items that generate greater satiation and satiety. In this study, we analyzed the dietary patterns of both HU and normal-weight subjects using a 7-day photographic food diary and found a clear discrimination in the dietary patterns between the two groups.

The estimated energy intake of the HU subjects from the photographic records were about 25% lower than the normal-weight group. This was a greater difference than we found using DLW. Given the accuracy of the method we used in terms of the weights of foods in the photographs (Supplementary Materials), this seems unlikely to be a consequence of error in the method at the level of weights of components of the individual meals. Nevertheless, the intakes of both groups were substantially lower than the corresponding predicted total energy expenditures by DLW (Figure S7), suggesting that the method did not capture all their intake. Under-reporting is also characteristic of methods based on the self-reporting of food intake, such as 24 h recall, food logging, or food frequency questionnaires (FFQ), which are simple and cheap to administer but have poor validity for most nutrients [18,19,20,21,22]. Image-based dietary records can collect detailed dietary intake information and reduce the burden on subjects [23,24]. It is apparent that the photographic record is more accurate than the 24 h recall and questionnaires, and the estimation of portion size in this study is considered to be reliable. However, our data suggest that photographing all consumed food, while superficially more objective than recall methods, may also be prone to under-reporting. There are two potential reasons for this under-reporting. First, subjects might not photograph all the foods they consumed, for example, omitting snacks. Second, the weight of the food may have been accurately captured (as in the validation study) but an error may have been introduced because the specific energy contents of different foods was not known. In the current context, this may be exacerbated by the complexity of cooking in the Chinese diet and the addition of a lot of oil and flavorings in the process, making it difficult to convert from weights of food to their calorie equivalents. Future studies need to explore the errors introduced because of variation in the energy density of specific foods. Moreover, while the photographic diary approach is an efficient way to potentially identify the energy and macronutrient intake of people, with a bearable burden on participants, it is really time consuming to analyze. The development of AI-based algorithms to speed up the image analysis may be a useful development in this method in the future [25].

The HU individuals exhibited a dietary pattern characterized by a higher percentage consumption of rice and vegetables, and a lower percentage intake of livestock meat, poultry, and starchy roots. Consequently, HU individuals had a higher percentage of energy derived from carbohydrates and a lower percentage from fats compared to the normal-weight population. Additionally, the HU population tended to favor plant protein over animal protein. Previous studies have identified total energy intake as an important factor driving weight gain [26], while others have focused on the contributions of specific macronutrients [27,28]. In addition to total energy intake, the macronutrient composition, namely the percentage of energy intake from carbohydrates, proteins, and fats, potentially have an important influence on energy balance [29]. There is a long history of debate on the effects of fat, carbohydrate, and protein on body weight management [30,31,32]. However, controversy still remains as to whether low-fat or low-carbohydrate diets are more conducive to weight loss [33]. Several studies have shown that energy-restricted dietary approaches to weight loss, which include low-fat, low-carbohydrate, moderate-to-high-protein, and macronutrient-targeted diets, all lead to similar clinically significant weight loss after 6 months, or 1 or 2 years [31,34,35]. The conventional energy balance model of obesity suggests that excess total energy intake leads to circulating excess metabolic fuel, and energy is stored in the form of fat [36]. An increase in the dietary portion of fat, which has the highest energy density, is closely associated with obesity [37,38,39]. However, in recent years, some researchers have proposed the carbohydrate–insulin obesity model, which suggests that consuming a high intake of carbohydrates causes an increase in insulin secretion, promotes fat synthesis and storage, and reduces the amount of metabolic fuel available in the circulation, which induces hunger and increases food consumption. This suggests that an increase in high-glycaemic-index carbohydrates is closely related to obesity [40,41,42]. In our study, however, we found that the HU population maintained low body weights despite having a dietary pattern of higher carbohydrate intake than the normal-weight population. However, it was not possible to ascribe accurate estimates of glycaemic index to the consumed foods, and hence this high carbohydrate intake might still be consistent with predictions of the CIM. Healthy underweight subjects did have considerably less fat intake as part of their lower overall energy intake, consistent with the energy balance model explaining the development of obesity [37,38,39]. Our data are consistent with previous studies suggesting that fat intake is more closely related to body weight [43].

Moreover, the healthy underweight subjects had more plant proteins and less animal proteins than the normal-weight subjects, although there was no difference in the total protein intake. Previous studies have demonstrated that a plant-based low-fat diet has a higher glycemic load compared to an animal-based ketogenic low-carbohydrate diet, resulting in higher postprandial blood glucose and insulin levels, but overall energy intake is spontaneously reduced, and participants lose weight and body fat [43]. The dietary pattern of the healthy underweight subjects matches the plant-based low-fat dietary pattern. In the plant-based dietary pattern, the proportion of fiber intake by the healthy underweight population was higher than the normal-weight subjects, which could potentially lead to higher satiety and lower energy intake [44,45,46]. However, the predicted satiety of the two diets was not different between the HU and normal-weight groups. Additionally, it is important to note that in this cross-sectional study, we cannot separate cause and effect. The diets may have contributed to their low body weights or their low body weights may have driven their diet choices.

## 5. Conclusions

In summary, the estimated energy intake from photographic food records of healthy underweight subjects was 25% lower than in normal-weight subjects. The lower energy intake of the HU population was correlated with higher carbohydrate and lower fat intake. Although there was no difference in protein intake, healthy underweight subjects ate less animal protein than plant protein. The dietary pattern of the HU population was characterized by a lower overall intake but a higher % intake of rice and vegetables and lower % intake of livestock and poultry meat, in comparison to the normal-weight subjects.

## Figures and Tables

**Table 1:** Basic characteristics of the participants.

	HU (*N* = 52)	Normal (*N* = 50)	*p* Value
Gender (F:M)	41:11	35:15	
Age, years	26.5 ± 3.3	26.1 ± 3.8	0.268
Height, cm	164.5 ± 8.5	167.0 ± 8.1	0.070
Weight, kg	46.2 ± 5.1	63.55 ± 7.0	<0.001
BMI, kg/m^2^	17.1 ± 1.0	22.7 ± 1.0	<0.001
Female			
Age, years	26.5 ± 3.1	26.1 ± 4.1	0.327
Height, cm	161.5 ± 5.6	162.9 ± 5.1	0.123
Weight, kg	44.7 ± 3.7	60.5 ± 4.9	<0.001
BMI, kg/m^2^	17.1 ±1.0	22.7 ± 1.1	<0.001
Male			
Age, years	26.6 ± 4.0	26.0 ± 3.0	0.325
Height, cm	175.9 ± 8.1	176.7 ± 4.9	0.384
Weight, kg	51.9 ± 5.9	70.7 ± 5.8	<0.001
BMI, kg/m^2^	16.7 ± 1.2	22.6 ± 1.0	<0.001

**Table 2:** Average weight of different food items consumed in the daily diet of HU and normal-weight groups.

	HU	Normal	*p* Value
Rice (g)	85.2 ± 54.5	73.0 ± 54.4	0.131
Flour (g)	138.8 ± 88.6	163.5 ± 60.6	0.052
Coarse food grains (g)	11.6 ± 15.5	20.7 ± 28.8	0.024
Beans (g)	3.8 ± 7.5	3.2 ± 6.3	0.345
Starchy roots (g)	11.2 ± 12.7	30.8 ± 26.1	<0.001
Vegetables (g)	110.3 ± 52.2	117.6 ± 59.1	0.253
Fruits (g)	45.0 ± 56.1	75.4 ± 77.9	0.013
Fungi and algae (g)	12.5 ± 15.3	13.4 ± 12.5	0.369
Livestock meat (g)	39.9 ± 22.3	77.2 ± 34.7	<0.001
Poultry meat (g)	13.6 ± 14.5	39.0 ± 29.6	<0.001
Aquatic products (g)	13.7 ± 16.0	15.6 ± 15.0	0.274
Eggs (g)	27.1 ± 21.4	31.7 ± 26.6	0.168
Milk and milk products (g)	51.1 ± 56.9	92.7 ± 94.4	0.004
Soy products (g)	16.6 ± 16.1	32.6 ± 29.8	<0.001
Nuts and seeds (g)	11.6 ± 16.1	18.0 ± 19.6	0.036
Drinks (g)	49.6 ± 72.5	96.6 ± 85.0	0.002

Note: Data are expressed as mean ± SD. Statistical analysis was performed using unpaired two-tailed Student’s *t* test.

**Table 3:** The energy intake and nutritional composition consumed at different times of the day, at breakfast, lunch, and dinner, respectively.

	HU	Normal	*p* Value
**Daily total**			
Energy, kcal	1138.3 ± 497.0	1526.2 ± 327.2	<0.001
Protein, g	73.6 ± 68.9	90.6 ± 52.4	0.166
Protein, %EI	23.6 ± 9.8	23 ± 8.8	0.326
Carbohydrates, g	142.3 ± 53.9	178.8 ± 42.6	<0.001
Carbohydrates, %EI	51.5 ± 8.0	47.4 ± 7.9	0.010
Fat, g	30.5 ± 11.2	49.8 ± 12.7	<0.001
Fat, %EI	24.6 ± 6.1	29.6 ± 5.3	<0.001
**Breakfast**			
Energy, kcal	212.0 ± 125.0	407.0 ± 190.3	<0.001
Protein, g	8.8 ± 4.9	23.0 ± 25.3	<0.001
Protein, %EI	17.8 ± 6.0	20.5 ± 11.5	0.146
Carbohydrates, g	27.6 ± 20.2	50.8 ± 24.7	<0.001
Carbohydrates, %EI	49.3 ± 18.0	50.6 ± 13.0	0.681
Fat, g	7.4 ± 4.9	12.4 ± 5.6	<0.001
Fat, %EI	32.9 ± 14.1	28.9 ± 10.3	0.115
**Lunch**			
Energy, kcal	458.8 ± 293.8	582.9 ± 173.6	0.011
Protein, g	33.0 ± 56.0	36.3 ± 25.9	0.711
Protein, %EI	23.5 ± 14.6	23.5 ± 9.2	0.994
Carbohydrates, g	56.5 ± 26.4	65.4 ± 20.4	0.063
Carbohydrates, %EI	52.9 ± 12.1	45.8 ± 9.5	0.001
Fat, g	11.2 ± 5.6	19.6 ± 6.4	<0.001
Fat, % EI	23.5 ± 7.6	30.7 ± 6.4	<0.001
**Dinner**			
Energy, kcal	467.6 ± 190.2	560.8 ± 160.9	0.009
Protein, g	31.8 ± 33.9	32.7 ± 24.5	0.879
Protein, %EI	23.9 ± 14.5	22.2 ± 10.7	0.507
Carbohydrates, g	58.1 ± 21.5	65.7 ± 20.8	0.071
Carbohydrates, %EI	51.7 ± 11.6	48 ± 10.9	0.102
Fat, g	12.0 ± 5.0	18.6 ± 8.0	<0.001
Fat, %EI	24.4 ± 7.7	29.8 ± 8.4	0.001

Note: Data are expressed as mean ± SD. Statistical analysis was performed using unpaired two-tailed Student’s *t* test.

## Data Availability

The data presented in this study are available on request from the corresponding author. The data are not publicly available due to ethical restrictions.

## Supplementary Materials

The following supporting information can be downloaded at: https://www.mdpi.com/article/10.3390/nu16213637/s1, Table S1. Differences in the actual weights and the estimated values from photographs by a dietician. Table S1. Differences in the actual weights and the estimated values from photographs by a dietician. Table S2. Energy intake and nutritional composition of the diets in different sex. Figure S1. Schematic overview of dietary assessment. (A) An outline of the main methods of dietary assessment. (B) Model for food recognition and analysis. Figure S2. The result of Elbow Method. Figure S3. Comparative analysis of weighed and estimated values for food portion sizes in a validation study. (A) Bland–Altman plot showed the comparison between weighed and estimated values. (B) Correlation between weighted and estimated values for food portion sizes. Black dots represent individual food items and hollow dots represent whole meals. Figure S4. Average frequency of different food items consumed in the daily diet of HU and normal-weight groups. Statistical analysis was performed using unpaired two-tailed Student’s *t* test. *, *p* < 0.05; **, *p* < 0.01; ***, *p* < 0.001. Figure S5. Satiety score of HU and normal-weight groups. Statistical analysis was performed using unpaired two-tailed Student’s *t* test. Figure S6. Correlation between estimated energy intake and predicted energy intake from an equation based on DLW measurements. The line shows the line of equality. On average, the estimated intakes of both groups based on the photographs were lower than the predicted intakes based on DLW. Figure S7. Importance ranking of food items using Random Forest Analysis. This figure illustrates the relative importance of different food items in predicting the diet type. Food items are ranked according to their Mean Decrease in Accuracy, which measures its impact on the model’s predictive power. Figure S8. RDA of food items on different dietary pattern clustering. Dark blue arrows represent food items. Green lines represent cluster 1, orange lines represent cluster 2 and purple lines represent cluster 3.
