# Supplementary material for: Dietary Patterns of Healthy Underweight Individuals Compared to Normal-BMI Individuals Using Photographic Food Diaries
Source: Nutrients. 2024 Oct 25;16(21):3637. doi: 10.3390/nu16213637 (PMC11547498; doi:10.3390/nu16213637)
Supplement: Supplementary file 1 [file nutrients-16-03637-s001.zip › nutrients-3248387-supplementary.pdf]

nutrients

Supplementary Materials

Dietary Patterns of Healthy Underweight Individuals Compared to Normal-BMI Individuals Using Photographic Food Diaries

Table S1. Differences in the actual weights and the estimated values from photographs by a dietician.

	1	2	3	4	5	6	7	8	9	10
Food item 1	9.43%	0.70%	9.87%	9.83%	1.07%	13.14%	8.79%	4.16%	2.94%	3.85%
Food item 2	7.05%	4.83%	5.56%	6.18%	1.86%	5.44%	1.55%	7.82%	11.42%	6.82%
Food item 3	4.00%	7.89%	0.53%	10.32%	14.70%	6.48%	5.98%	7.82%	5.74%	9.19%
Food item 4	4.37%	10.48%	14.59%	1.72%	0.20%	14.41%	0.53%	5.57%	11.67%	4.12%
Food item 5	0.85%	1.19%	6.22%	6.28%	10.60%	0.85%	4.12%	13.02%	7.44%	2.66%
**Whole meal**	**2.81%**	**1.50%**	**0.75%**	**0.87%**	**3.87%**	**3.08%**	**3.47%**	**0.75%**	**3.88%**	**0.71%**

Table S2. Energy intake and nutritional composition of the diets in different sex.

	HU	Normal	p value
**Female**			
Total energy, kcal	1070.6 ± 476.1	1514.9 ± 319.6	< 0.001
Carbohydrates, %EI	50.9 ± 8.2	47.5 ± 7.8	0.003
Protein, %EI	23.1 ± 9.7	22.6 ± 9.1	0.405
Fat, %EI	26 ± 5.6	29.9 ± 5.4	0.001
**Male**			
Total energy, kcal	1396.5 ± 487.5	1552.6 ± 354.3	0.176
Carbohydrates, %EI	53.2 ± 7.5	47.0 ± 8.1	0.028
Protein, %EI	24.9 ± 10.1	24.0 ± 8.2	0.4
Fat, %EI	21.9 ± 4.6	29.0 ± 5.2	< 0.001

Note: Data are expressed as mean ± SD. Statistical analysis was performed using unpaired two-tailed student's t test.

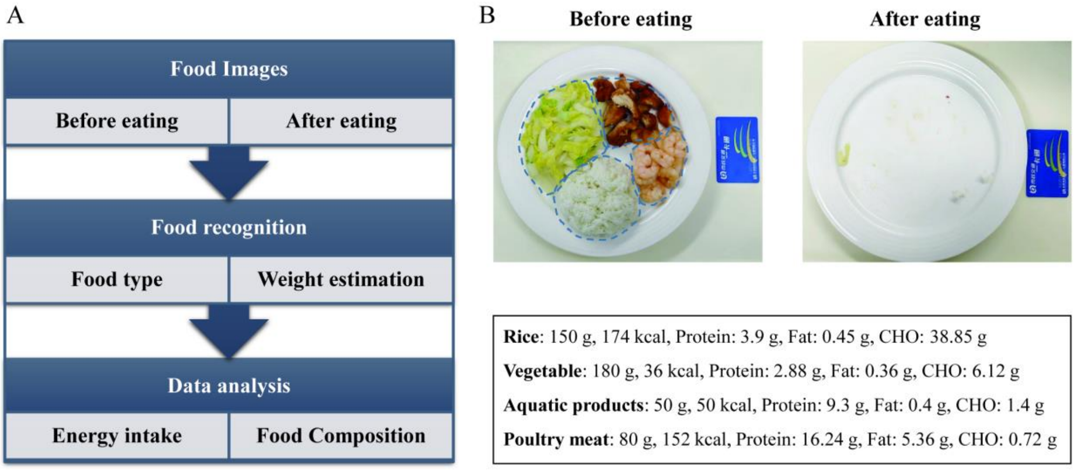

Figure S1. Schematic overview of dietary assessment. (A) An outline of the main methods of dietary assessment. (B) Model for food recognition and analysis.

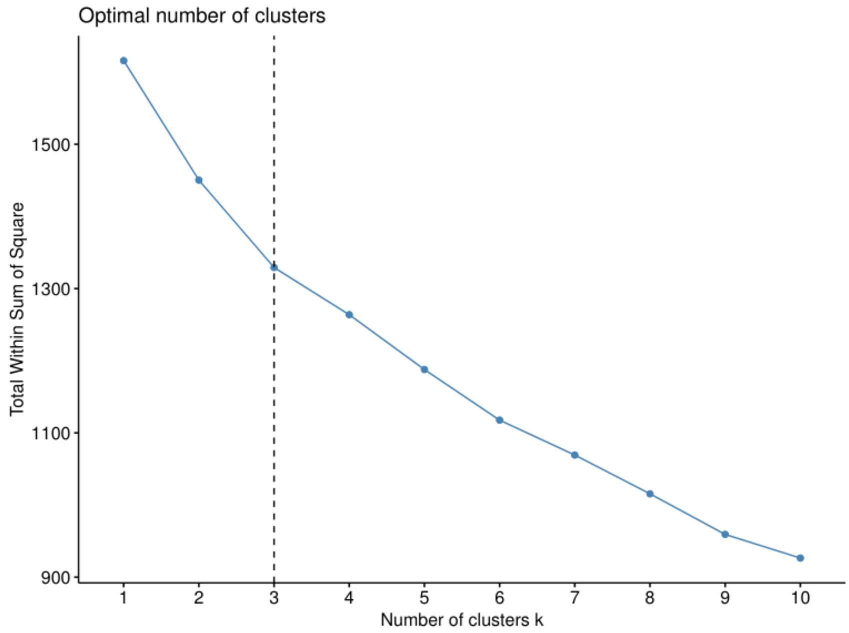

Figure S2. The result of Elbow Method.

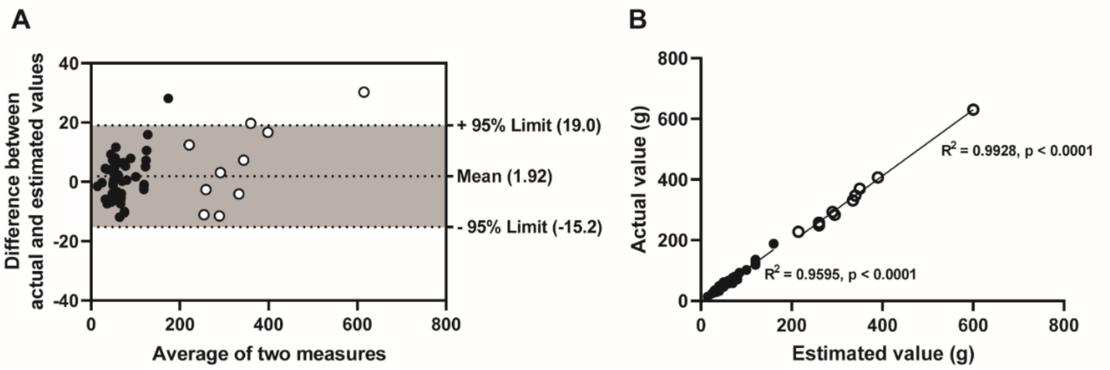

Figure S3. Comparative analysis of weighed and estimated values for food portion sizes in a validation study. (A) Bland-Altman plot showed the comparison between weighed and estimated values. (B) Correlation between weighted and estimated values for food portion sizes. Black dots represent individual food item and hollow dots represent whole meal.

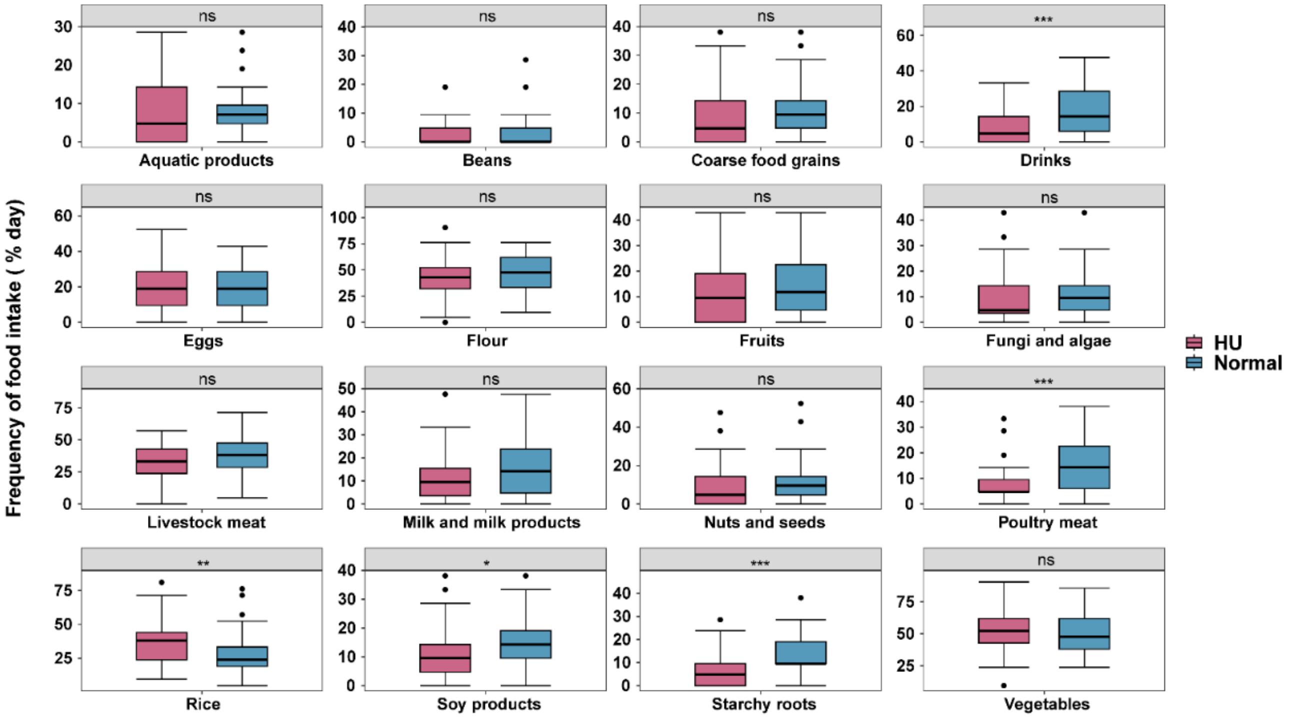

Figure S4. Average frequency of different food items consumed in the daily diet of HU and normal groups. Statistical analysis was performed using unpaired two-tailed student's t test. *, $p < 0.05$; **, $p < 0.01$; ***, $p < 0.001$.

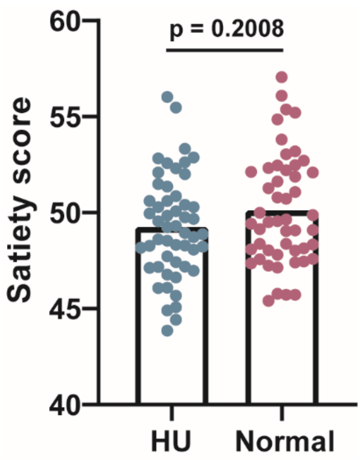

Figure S5. Satiety score of HU and normal groups. Statistical analysis was performed using unpaired two-tailed student's t test.

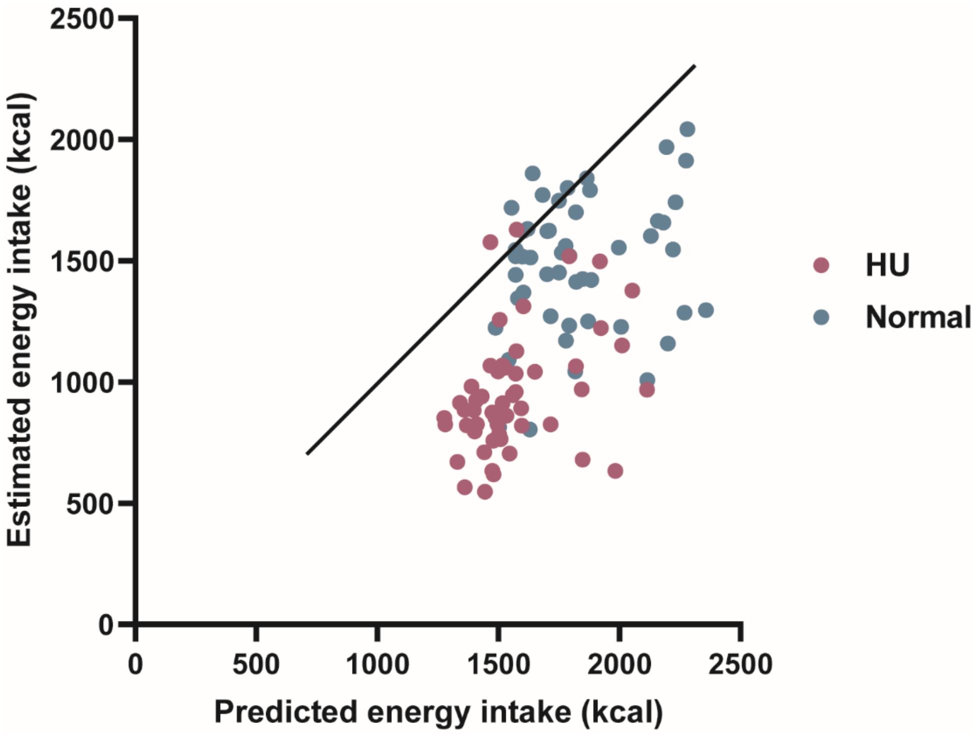

Figure S6. Correlation between estimated energy intake and predicted energy intake from an equation based on DLW measurements. The line shows the line of equality. On average the estimated intakes of both groups based on the photographs were lower than the predicted intakes based on DLW.

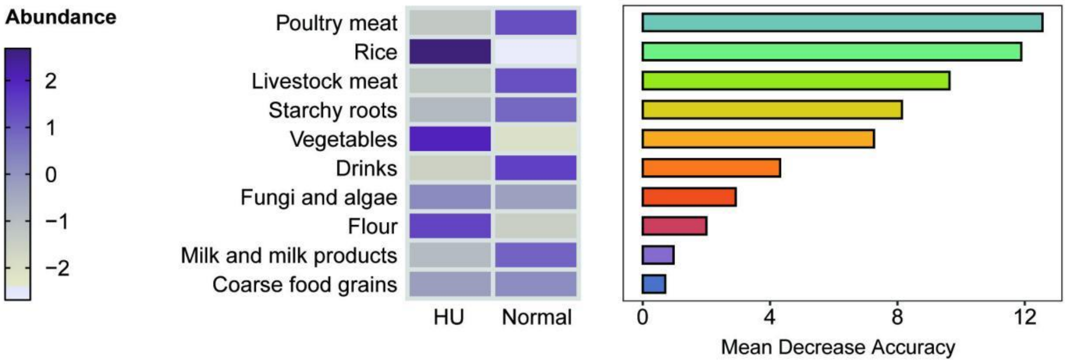

Figure S7. Importance ranking of food items using Random Forest Analysis. This figure illustrates the relative importance of different food items in predicting the diet type. Food items are ranked according to their Mean Decrease in Accuracy, which measures its impact on the model's predictive power.

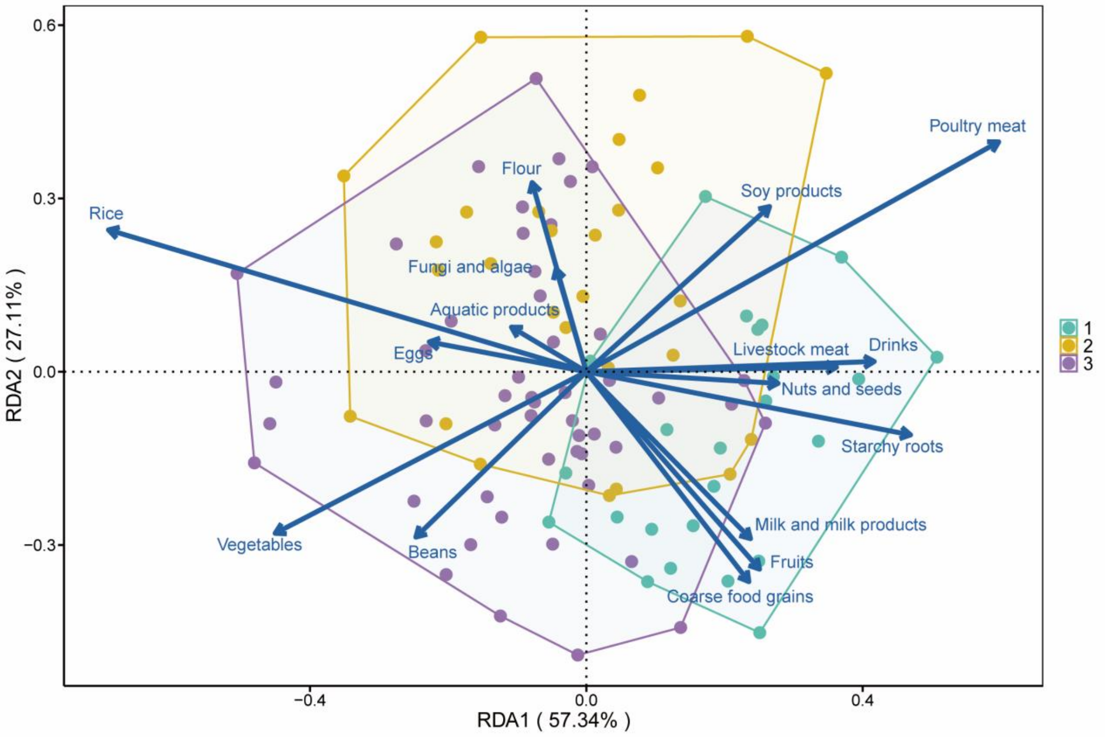

Figure S8. RDA analysis of food items on different dietary pattern clustering. Dark blue arrows represent food items. Green lines represent cluster 1, orange lines represent cluster 2 and purple lines represent cluster 3.
